# Letters to the editor on the Zika virus: a bibliometric analysis

**DOI:** 10.5195/jmla.2021.903

**Published:** 2021-04-01

**Authors:** Frances A. Delwiche

**Affiliations:** 1 Frances.Delwiche@uvm.edu, Library Associate Professor Emerita, Dana Medical Library, University of Vermont, Burlington, VT

**Keywords:** bibliometrics, bibliometric analysis, Letters to the Editor

## Abstract

**Objective:**

To conduct a bibliometric analysis of Letters to the Editor published on the Zika virus from 1952–2018.

**Methods:**

A PubMed search was conducted using the terms (Zika OR ZIKV). Results were limited to 1952–2018 and Publication Type = Letter. Results were exported to EndNote, and the full text of each Letter examined. Each Letter was assigned to one of five categories: Reader Response, Author Reply, Observation, Case Report, or Research. Additional study parameters included number of authors, number of references, use of graphics, and funding. Citation reports were generated for each category and the entire dataset, producing lists sorted by Times Cited.

**Results:**

Of 499 Letters, only 15 (3.0%) were published before 2016. In 2016, at the height of the Zika virus epidemic in the Americas, 244 (48.9%) Letters were published, dropping to 145 (29.1%) in 2017 and 95 (19.0%) in 2018. Letters included 149 (29.9%) Reader Responses, 56 (11.2%) Author Replies, 112 (22.4%) Observations, 70 (14.0%) Case Reports, and 112 (22.4%) Research. The Letters were written by 1–35 authors; 369 (74.0%) Letters had 1–5 authors, and 130 (26.0%) had 6 or more. The Letters cited 0–63 references, with an average of 7.0 per Letter. Graphics appeared in 192 (38.5%) Letters, and 77 (15.4%) Letters reported funding. An interesting anomaly was the 104 (20.8%) Letters authored or co-authored by 1 individual.

**Conclusion:**

Letters to the Editor remain an important component of scientific communication and may serve as a valuable source of clinical and research information.

## INTRODUCTION

Letters to the Editor (“Letters”) are a classic but often overlooked and underappreciated component of scholarly communication in the biomedical sciences. Unlike original research articles, which typically follow the IMRaD format (Introduction, Methods, Results, and Discussion), Letters do not have a standardized format, structure, or layout. Their length varies widely—they may be as brief as six sentences [1] or as long as six pages [2] or more. They are frequently written in the form of traditional correspondence, opening with a salutation such as “Sir” or “To the Editor”, and “signed” by one or more authors. Although addressed to the editor, the intended audience is the entire journal readership. Letters published by journals that are indexed by MEDLINE are assigned MeSH headings and the publication type Letter.

Letters to the Editor generally fall into one of five categories, based on their content. The first category, Reader Response, may be defined as a Letter that addresses the content of a specific article that was previously published in the same journal. It may have been written to praise the original article, to question or criticize some aspect of it, or to offer additional information that expands the discussion. The second category, Author Reply, is a direct response by the author(s) of the original article to the comments and/or criticisms made in the Reader Response. These two types of Letters serve to fulfill the most basic function of Letters to the Editor, which is to encourage discussion and debate among the journal's readers, thus increasing engagement with the publication [3–4]. Depending upon the level of reader interest, some journal editors receive more Letter submissions than they are able to publish [5], while other editors lament the dearth of Letters, prompting pleas for greater participation [6].

A second and far more important function of these two types of Letters is to serve as a form of post-publication peer review in which readers have the opportunity to raise questions, concerns, or criticisms of published papers [3, 7–8], thus playing “an important corrective role in science” [9]. For example, Letters written in response to randomized controlled trials (RCTs) frequently raise important questions regarding the methodology employed, especially relating to the population, intervention, and analysis [10]. Issues raised in a Letter may ultimately lead to the retraction of the original article [11–12], and occasionally a Letter is used to announce the voluntary retraction of a paper [13].

Some have argued that authors of Letters “have more credibility than … prepublication peer-reviewers because the former opinions are published and signed” [14]. Unlike informal means of scientific communication, such as social media, blog posts, or personal conversations, Letters published in journals that are indexed by bibliographic databases become part of the permanent scientific record [3, 15]. However, in order for the record to be complete, published comments, as well as the author replies, must be linked to the original article in bibliographic databases; without that, “… the impact of the paper endures, while its relevant comment, which may be crucial to the paper's interpretation and relevance, does not” [8].

The other three categories of Letters—Observations, Case Reports, and Research—are not directly associated with a prior article, although they may refer to one to establish context. Letters categorized as Observations are written with the intent to inform, educate, or stimulate discussion. They offer opinions or commentaries, provide topic reviews, share anecdotal findings, or summarize previous data. They are sometimes quite lengthy and may contain images and graphics, but they do not convey original findings. This category includes all Letters that do not fit into any of the other four categories.

Letters categorized as Case Reports provide a summary of one or more patients, with a description of symptoms, diagnosis, treatment, and/or outcome, followed by a discussion of the unique aspects of the case that warrant publication. In MEDLINE, case reports published as Letters are usually assigned both the Letter and Case Report publication type.

Finally, Letters reporting the results of original investigations are categorized as Research. They contain “original research … which may lack sufficient content to warrant a full-length manuscript but at the same time cannot be ignored …” [8]. Research Letters are typically small, narrowly focused studies, such as early phase clinical trials, pilot studies, study protocols, or preliminary research findings [16]. They generally employ a straightforward methodology, use readily referenced data sources, and apply uncomplicated statistical analyses [17]. One journal describes Research Letters as “the most prestigious form of correspondence”, referring to them as “the seed-corn of research” from which more substantial research studies may germinate [18]. Occasionally, journal editors reject a research manuscript submitted for publication as a full paper, with the recommendation that the authors revise and resubmit it as a Letter [7]. Case reports and original research are so commonly published as Letters that some journals have created dedicated columns in their publications for these types of Letters [16–17, 19–20].

The International Committee of Medical Journal Editors (ICMJE) requires that medical journals provide readers with a means through which they can respond to published articles and, furthermore, specifies that the authors of the original articles have an obligation to respond to substantive criticisms [21]. However, editors of medical journals have a great deal of latitude in how they fulfill the ICMJE requirement, allowing them to dictate the form and content of Letters included in their publications. Due to space constraints (often a remnant of print publishing), many journals impose limits on the number of words, authors, references, and figures allowed [5, 7]. Some journals accept only Letters written in response to articles published in that journal, often with time restrictions following publication of the original article [6–8], while others accept Letters on any relevant topic, at any time.

The degree to which Letters are subjected to pre-publication peer review depends upon the journal's editorial policies, and may range from traditional external peer review to a more limited review by the editor. Journals may have different policies for different types of Letters, with Case Reports and Research Letters typically being subjected to full peer review, while Reader Responses receive only a limited review [17, 22–24]. Since journal editors are usually experts in their fields, a review conducted solely by the editor can nevertheless be considered a form of peer review [24].

Letters have an educational and career development function as well, often serving as a means by which new members of a discipline can break into the realm of scholarly writing and publishing [4, 7, 15]. Letter writing as a group activity has been used by journal clubs for medical students [25] and hospital faculty and fellows [26] as a way of teaching critical appraisal and scientific writing skills. Tierney, O’Rourke, and Fenton maintain that writing an accurate critique of a research article in the form of a Letter can be challenging, and is an activity that should be encouraged and given more recognition [8]. Letters are frequently published on a shorter turnaround time than full articles [5] and thus are an attractive option for authors who wish to have their material published expeditiously [20].

Previously published bibliometric studies on Letters to the Editor in the biomedical sciences fall into one of four categories. The first category consists of studies that focus on Letters published by one particular journal. Examples of studies in this category include Boyton and Arnold's 1990 study of *BMJ* [27], Caswell's 1991 study of the *Medical Journal of Australia* [28], and Rosell Pradas and Lacasaña Navarro's 2007 study of *Farmacia Hospitalaria* [29]. The studies in this category are of particular interest to the editors, editorial boards, and reading audiences of those journals, but their findings may have limited generalizability.

A second category of bibliometric study examines Letters published by a small group of journals. The classic 1983 study by Spodick and Goldberg reviewed a sample of Letters published by four general medical journals (*BMJ, JAMA, Lancet*, and *New England Journal of Medicine*) and four medical specialty journals [30]. More recently, Von Elm, Wandel, and Jüni examined all Letters published in 2002 and 2007 by eight general medicine/internal medicine journals, including all four general medical journals from the earlier study [31]. Lastly, a 2015 study by Tierney, O’Rourke, and Fenton analyzed all Letters published by four leading otorhinolaryngology journals [8]. Findings from these studies may have applicability to similar journals in the same or closely related disciplines.

A third category of bibliometric study focuses on the content of Letters written in response to published research articles. Horton's 2002 study looked at all Letters published in *The Lancet* in response to three RCTs on hypertension [32]. The author catalogued the criticisms, comments, and questions raised in the Letters, analyzed the author responses, and evaluated subsequently published practice guidelines to determine whether they incorporated the issues raised in the Letters. In 2010, a cohort study by Gotzsche et al. analyzed author responses to reader criticisms of published research papers that were posted in *BMJ's* online comment section [33]. A 2013 study by Kastner et al. analyzed the content of Letters written in response to 175 RCTs published in 5 high-impact medical journals [10].

The last category of bibliometric study consists of Letters that serve as a source of data for scholarly research. In 2008, Anthony and Barkell analyzed the content of Letters published in a prominent nursing journal from 1900 to 2005 as a means of understanding nurses’ professional concerns [34]. A 2012 study by Yang, Srinivasan, and Polgreen focused on Letters as a source of information on adverse drug events [35]. And in 2016, a study by Chauhan et al. investigated the risk of bias in RCTs published as Letters versus that in RCTs published as full papers [36].

Several bibliometric studies have been conducted on infectious diseases in general, but they either excluded Letters altogether [37–39] or did not analyze them as a separate category [40]. A number of bibliometric studies have been conducted on the Zika virus specifically [41–46], but none addressed Letters separately. One of these grouped Letters with editorials and commentaries in order to compare the number of “opinion pieces” with the number of “research articles” on six infectious diseases, including Zika virus disease [41]. Bibliometric studies have been conducted on other arboviral diseases, such as chikungunya [47], dengue [48–49], Oropouche [50], and Venezuelan equine encephalitis [51]. Of these, only one study on dengue mentioned Letters, noting that they constituted 4.31% of the total articles published [49]. More recently, three bibliometric studies on COVID-19 listed the percentage of total publications composed of Letters as 3.7% [52], 7.1% [53], and 16.4% [54].

These studies are illustrative in their own right, but because of their narrow focus none were able to provide a general characterization of Letters in the biomedical sciences. Therefore, this study aimed to examine a representative sample of Letters published on an interdisciplinary topic in a wide variety of biomedical and health sciences journals, with the ultimate goal of constructing a broad picture of the nature of Letters.

## METHODS

The first step of the study was to select a sample topic. Requirements for the sample topic were that it (1) be relevant to multiple disciplines, (2) have a body of literature small enough to be searched comprehensively in a major biomedical journal literature database, and (3) that its literature be readily accessible. The topic of the Zika virus easily satisfied all three criteria. Owing to the extensive outbreak in 2015–2016 in the Americas, it was of interest to researchers in many pre-clinical sciences and medical/nursing disciplines, including medicine, infectious disease, virology, immunology, neurology, obstetrics, pediatrics, and public and global health. However, before 2016 the level of interest in the Zika virus was relatively low, and consequently the body of literature on the Zika virus was extraordinarily small, with only 139 articles published prior to that date [41]. Therefore, it was likely that the majority of Letters on this topic would have been published in 2016 or later, and the full text would be easily obtained.

The raw data for this study was obtained from PubMed, the U.S. National Library of Medicine's premier database for biomedical literature. PubMed was chosen because of its excellent worldwide reputation, broad subject coverage from the pre-clinical sciences to the clinical specialties, and free access via the Internet. PubMed encompasses the entire MEDLINE database as well as several smaller subsets of documents that exist outside of MEDLINE. Citations in MEDLINE derive from more than 5,200 journals published worldwide in about 40 languages [55].

In this study, a Letter to the Editor was defined as an article about the Zika virus or Zika virus infection that was published in the Letters or Correspondence column or section of a scholarly journal. A PubMed search was conducted on October 17, 2018 using the terms zika[All Fields] OR zikv[All Fields]. The results were limited to Entrez Date = 1952–2017 and Publication Type = Letter. The PubMed search was then repeated using the same search terms and Entrez Date range, but this time limiting to Publication Type = Comment. Articles resulting from this search that met the above definition of a Letter were added to the dataset, as were Letters discovered manually when reviewing PDFs of other Letters. On July 15, 2019, the process was repeated for calendar year 2018. Although additional records for 2018 may have been added to the database after that date, the numbers are likely to be small and their omission expected to have negligible impact on the final results.

The PubMed search results were exported to an EndNote library, de-duplicated, and the full-text of each Letter obtained. The results were exported from EndNote to a Microsoft Excel spreadsheet, where data for all parameters were entered and analyzed. Throughout the process, multiple cross-checks were conducted to ensure that data were accurately transcribed. The content of the Letters was reviewed manually, and each Letter assigned to one of the five previously described categories: Reader Response, Author Reply, Observation, Case Report, or Research.

Additional data collected included publication date, number of authors, number of references, use of graphics, and funding. Data for the first three parameters were in numerical form, while the last two were a dichotomous Yes/No. Graphics included any non-text content within the article, such as tables, charts, diagrams, photographs, or radiographic images. Funding referred to financial support from internal or external sources. Finally, as a measure of the usage of the Letters by the scientific community, citation reports from Web of Science were generated on July 29, 2019, for each of the five categories and for the entire dataset. The resulting lists were sorted by Times Cited, enabling the most highly cited Letters to be identified.

## RESULTS

**Figure 1:**
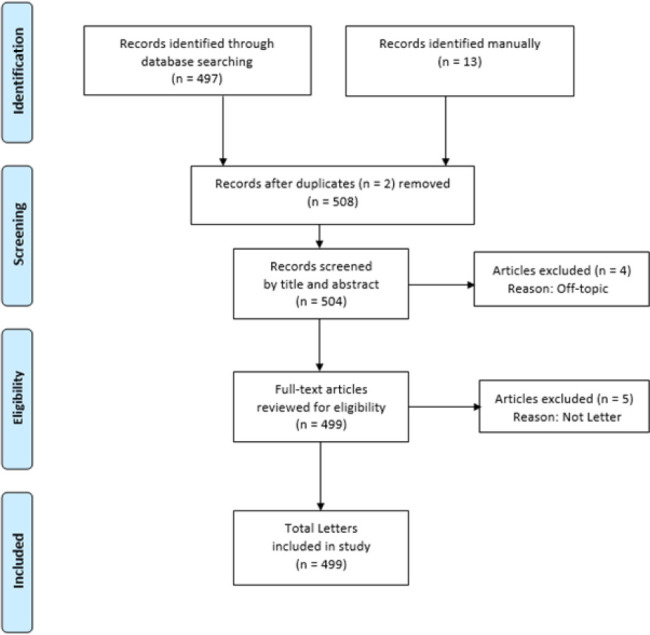
Source of Letters Included in Study. Adapted from Moher D, Liberati A, Tetzlaff J, Altman DG; PRISMA Group. Preferred reporting items for systematic reviews and meta-analyses: the PRISMA statement. PLoS Med. 2009 Jul 21;6(7):e1000097. doi: 10.1371/journal.pmed.1000097.

The PubMed searches brought up an initial set of 5,769 results on the Zika virus. Application of Entrez date and publication type limits yielded 497 Letters. After adding Letters discovered manually (n=13), removing duplicate Letters (n=2), and excluding records that were off-topic or that were not Letters (n=9), a final set of 499 Letters was obtained (Figure 1).

No Letters were published on this topic prior to 2012, and only 15 Letters (3.0%) were published between 2012 and 2015. In 2016, at the height of the Zika virus epidemic in the Americas, 244 Letters were published (48.9%). As the number of new cases diminished, the number of published Letters plummeted, dropping to 145 (29.1%) in 2017 and 95 (19.0%) in 2018.

**Figure 2:**
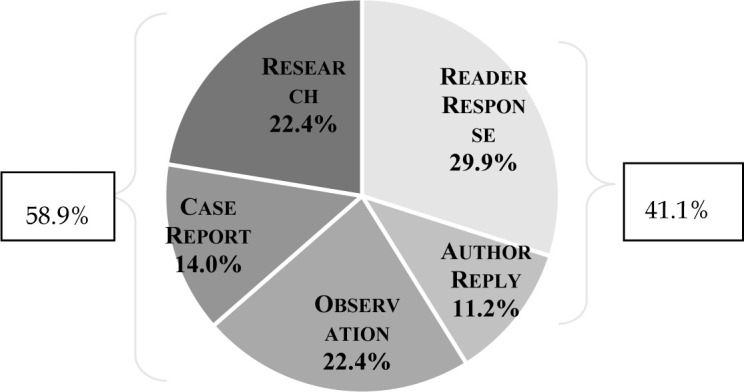
Categories of Letters*

The Letters were categorized as 149 (29.9%) Reader Responses, 56 (11.2%) Author Replies, 112 (22.4%) Observations, 70 (14.0%) Case Reports, and 112 (22.4%) Research (Figure 2). The percentage of Reader Responses receiving an Author Reply was 37.6%. The combined Reader Responses and Author Replies totaled 205 (41.1%), while the other three categories totaled 294 (58.9%).

* Total = 99.9% due to rounding

Among the 15 Letters published between 1952 and 2015, there was only one Reader Response and its associated Author Reply (6.7% each). In 2016, as the epidemic gained momentum and the total number of Letters increased dramatically, the percent of Reader Responses rose to 25.4%. In 2017 and 2018, Reader Responses comprised even greater percentages, at 36.6% and 34.7%, respectively (Table 1).

**Table 1:** Categories of Letters^*^

Category	1952-2015	2016	2017	2018	TOTAL
Reader Response	1 (6.7%)	62 (25.4%)	53 (36.6%)	33 (34.7%)	149 (29.9%)
Author Reply	1 (6.7%)	23 (9.4%)	17 (11.7%)	15 (15.8%)	56 (11.2%)
Observation	4 (26.7%)	66 (27.1%)	34 (23.4%)	8 (8.4%)	112 (22.4%)
Case Report	5 (33.3%)	42 (17.2%)	12 (8.3%)	11 (11.6%)	70 (14.0%)
Research	4 (26.7%)	51 (20.9%)	29 (20.0%)	28 (29.5%)	112 (22.4%)
TOTAL	15 (100%)	244 (100%)	145 (100%)	95 (100%)	499 (100.0%^*^)

* Percentage rounded up to 100

**Figure 3:**
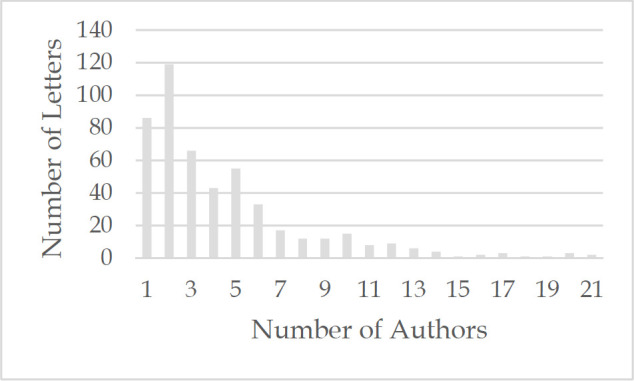
Number of Authors per Letter^*^

^*^ Excludes one Letter with 35 authors.

The average number of authors per Letter was 4.5 (range 1–35). The mode was 2, with 119 Letters (23.8%), followed by 86 Letters (17.2%) with 1 author and 66 Letters (13.2%) with 3 authors. There were 369 (74.0%) Letters with 1–5 authors, 124 (24.8%) Letters with 6–19 authors, and 6 (1.2%) Letters with 20 or more authors (Figure 3).

The Letters were written by 341 unique lead or sole authors. Although an analysis of author productivity was beyond the scope of this study, it was notable that 303 (88.9%) of the authors were lead or sole author on only 1 Letter, with only 38 (11.1%) being lead/sole author on more than 1 Letter. Among all authors, 24 (7.0%) were lead/sole author on 2 Letters, 6 (1.8%) were lead/sole author on 3 Letters, and 6 (1.8%) were lead/sole author on 5–10 Letters. However, there were two notable outliers. One individual, hereafter referred to as Author A, was sole author on 36 Letters, and a second author, referred to herein as Author B, was lead author on 49 Letters, all of which were co-authored by Author A. Altogether, Author A was sole author or 1 of 2 authors on 104 Letters, comprising 20.8% of the Letters in this study. Of these, 78 (75.0%) were Reader Responses, 25 (24.0%) Observations, and 1 (1.0%) Research.

**Figure 4:**
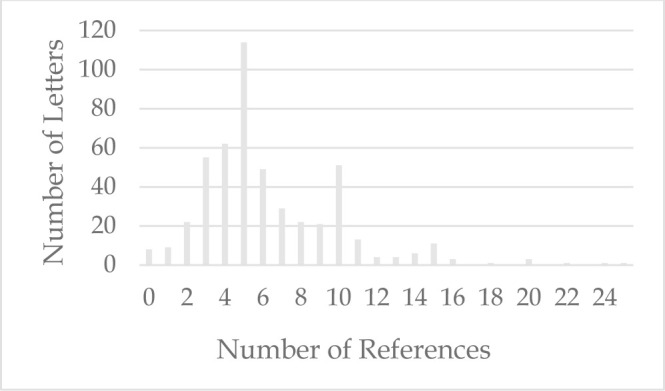
Letters with 0–25 References, N=490*

An average of 7.0 references were cited per Letter (range 0–63). Only 8 (1.6%) Letters did not cite any references; conversely, 9 (1.8%) Letters cited 26 or more references, including Letters citing 49, 51, and 63 references. The mode was 5 references, seen in 114 (22.8%) Letters, and 434 (87.0%) Letters cited 1–10 references (Figure 4).

* Excludes 9 Letters with 26–63 references

Graphics were included in 192 (38.5%) Letters. Of these, 180 (93.8%) were categorized as Observations, Case Reports, or Research. Predictably, many of these—101 (52.6%)—were categorized as Research.

Funding from internal or external sources was reported by 77 (15.4%) Letters. Again, most of these—72 (93.5%)—were categorized as Observations, Case Reports, or Research, including 47 (61.0%) categorized as Research.

Web of Science provided citation information for 493 (98.8%) Letters, which indicated they were cited a total of 7,823 times. Sorting by Times Cited revealed that 7 Letters were cited more than 200 times, 12 were cited 101–200 times, and 21 were cited 50–100 times. The highest-ranking Letter was cited 527 times in less than 4 years following its publication [56]. The 112 Research Letters received 3,220 (41.2%) citations, for an average of 28.8 citations/Letter. The 70 Case Reports received 3,144 (40.2%) citations, for an average of 44.9 citations/Letter. Together, the Case Reports and Research categories accounted for 6,364 (81.3%) of the citations. Citation information for 102 of the 104 Letters written by Author A showed that they were cited a total of 147 times, with 1 Letter cited 67 times, 1 cited 14 times, 34 cited 1–6 times, and 66 not cited.

## DISCUSSION

The study's findings were compared with those of previous studies that addressed the same parameters (Table 2). With solid representation in all 5 categories of Letters, this study shows that Letters continue to fulfill a vital function in scientific communication. The fact that Reader Responses and Author Replies together comprised more than 41% of the total provides evidence that Letters continue to promote scientific discourse and enable post-publication review, although the 38% response rate by authors to Reader Responses is slightly lower than in previous studies. More importantly, this study shines light on the key role of Letters in conveying clinical and research information, as 59% of the Letters fell into the Observation, Case Reports, or Research categories. This is substantially greater than the low of 35%, but well below the high of 72%, of the 3 previous studies reporting these data.

**Table 2:** Study Results Compared with Results from Previous Studies.

Parameter	Caswell [28]	Gotzsche et al. [33]	Horton [32]	Rosell Pradas & Lacasaña Navarro [29]	Tierney et al. [8]	Present Study
Letters not associated with a previous article	35%			72%	40/55%	59%
Reader Responses receiving Author Replies		45%	40%			38%
Mean number of authors				3.0	2.6	4.5
Mean number of references				5.7	3.6	7.0
Letters including graphics				24%	38%	39%

At 14.0%, the Case Report category was well represented in this study. For a clinical topic with a sparse knowledge base, case reports published as Letters can prove invaluable; this is especially true during an outbreak of an emerging disease, such as that caused by the Zika virus. Taken alone, case reports rank low on the hierarchy of levels of evidence, but when multiple cases are viewed together, they help establish a case definition and elucidate the natural history of the disease [57]. Cappell points out that case reports have both research value, in reporting novel findings that can lead to further studies, and educational value, in presenting rare clinical phenomena and reviewing the existing literature [23].

With 22.4% of the Letters in this study categorized as Research, it is clear that Letters serve as a rich source of original information. Zylke touts the value of research letters, noting that they often receive a substantial number of citations, garner national press coverage, and have even influenced federal drug policy [17]. Although RCTs are often published as Letters, Chauhan et al. found no evidence of a greater risk of bias with RCTs published as Letters; however, they did find that RCT results that were not statistically significant were more likely to be published as Letters than full papers [36]. RCTs published as Letters often show a smaller treatment effect than published trials, and therefore their inclusion in systematic reviews and meta-analyses can be an important strategy for minimizing publication bias [36, 58].

Some question the practice of publishing research as a Letter without full peer review, claiming, “Weak or incomplete research is worse than no research as it confuses the literature. [Furthermore], if published, it can be cited with seemingly the same validity as a full peer-reviewed article” [59]. Of course, the scientific literature is replete with examples of failures of the peer review system and flawed studies still may be published, even in the best medical journals [33]. Whether published as a full paper or research letter, findings from any original study must be critically appraised and utilized with care.

At 4.5, the mean number of authors for Letters in this study was higher than the figures previously reported. Of greater importance is the fact that 413 (82.8%) of the Letters had more than 1 author, and more than a quarter of them had 6 or more authors. These findings suggest that rather than reflecting the unconstrained views of one individual, the majority of Letters are the product of professional collaboration, which itself can serve as a form of internal quality control.

An interesting finding was the unusually large number of Letters (n=104) written by the individual referred to as Author A, either as sole author (n=36) or as 1 of 2 authors (n=68). In 2011, Neghina referred to a similar situation [60], which this author believes involved the same individual. Of the 104 Letters, 78 were Reader Responses, constituting 52.3% of Letters in that category. Furthermore, 27 (48.2%) of the Author Replies were to Reader Responses authored/coauthored by Author A. To assess the impact of this individual's Letters on the results of the overall study would require detailed content and author productivity analyses, which were beyond the scope of this study. Likewise, the contribution to the overall scientific discourse on the Zika virus made by this individual through their published Letters would require thorough analysis by subject experts.

The mean number of references cited in this study was slightly greater than that reported by two previous studies. Taking a broader view, the fact that 229 (45.9%) of the Letters listed more than 5 references suggests a recognition of the importance of backing up one's views with published evidence, especially if the Letter will not be subjected to full peer review.

The number of Letters in this study that included graphics was substantially higher than in one previous study and similar to that of another study. Inclusion of graphics may suggest increased complexity or novelty of content, requiring visuals to support the text. Tierney et al. argue that “diagrams … are only really applicable to original material not letters critiquing published works … [and] help to illustrate new concepts and ideas…” [8]. The present study supports that view, as over half of the Letters with graphics were categorized as Research.

The Web of Science Citation Reports indicate that Letters are indeed read and cited by members of the scientific community, occasionally quite frequently. For this topic and time period, Letters clearly had a place in the overall scholarly conversation, providing information deemed worthy of being cited by authors and researchers. A more granular analysis would be required to determine the degree of self-citation that occurred.

## LIMITATIONS

The data for this study were obtained from one database, PubMed. Since no database covers all published journals, it likely did not include all Letters published on the Zika virus. That said, PubMed is one of the largest and most comprehensive databases available for biomedical journal literature. It provides excellent coverage of a broad range of pre-clinical and clinical topics and is widely used internationally. For a more comprehensive study, data should be included from additional databases such as EMBASE, LILACS, SciELO, Scopus, and Web of Science.

Journal editorial policies may have impacted the study's findings. First, this study examined only published Letters. However, since journal editors often receive more Letters than the allotted space can accommodate, they are forced to make decisions as to which to publish, introducing the possibility of selection bias [33] or editorial censorship [10]. Second, editorial policies governing the length, content, or number of authors, references, or graphics must also be considered. Although this study no doubt included Letters from journals with such restrictions, the subject-based methodology provided a composite picture of Letters published by a wide range of journals.

Several limitations stem from the PubMed search strategy. Though it was intentionally broad, it would nevertheless be unable to retrieve records from which the search terms were missing, as could occur when incomplete indexing is coupled with an imprecise article title, such as “Reply”. Since Letters usually lack an abstract and may not be fully indexed initially, authors can enhance the discoverability of their Letters by carefully choosing a descriptive title that would ensure retrieval. Also, the search strategy relied upon MEDLINE's publication type indexing; however, an occasional Letter lacked the Letter publication type search tag, being initially indexed only as Journal Article [61] or Comment [62]. Von Elm et al. similarly found “that the assignment of publication types in PubMed was not always reliable” [31]. Rarely, a Letter was discovered via manual review that lacked a PubMed record altogether, never having been entered into the database [63–64].

Finally, the sample topic chosen for this study would inevitably influence the results to some degree. In this case, the urgency of the Zika virus epidemic may have inflated reader engagement with the published literature beyond typical levels. It may also have induced some researchers to publish important findings via a Letter rather than wait for the lengthier process required for a full paper. Nevertheless, as the first of its kind, this study provides a firm baseline for describing the general nature of Letters as a publication type.

## CONCLUSIONS

This study demonstrates that Letters are often much more than simply reader reactions to previously published articles; indeed, they frequently function as a source of valuable and timely clinical and research information. They often represent collaboration by multiple authors, are usually backed up by evidence from the literature, and are regularly cited by members of the scientific community. Thus, despite the advent of social media and other informal communication tools, this study provides evidence that formally published Letters remain an important element of scientific communication and possess the potential to make valuable contributions to the knowledge base.

## Data Availability

Data associated with this article are available in the Figshare repository at https://doi.org/10.6084/m9.figshare.11299244.v1 and https://doi.org/10.6084/m9.figshare.11299262.v1.
